# Post-Acute COVID-19 syndrome (PACS) right atrioventricular and vena cava thrombus on top of a myxoma. A Case report

**DOI:** 10.1186/s13019-022-01998-1

**Published:** 2022-10-08

**Authors:** Ghaliya Mohamed H Alrifae, Amel Ahmed Said Almuquddami, Khaled Masaud Etaleb, Mohamed Hadi Mohamed Abdelhamid

**Affiliations:** 1 Department of Cardiac Surgery, Tripoli University Hospital, Tripoli, Libya; 2 Department of Oncology, Tripoli University Hospital, Tripoli, Libya; 3 Head of Researchs and Sciences Committees Office, National Center for Disease Control (NCDC), Tripoli, Libya; 4 Department of Cell Biology and Tissue Culture, Biotechnology Research Center (BTRC), Tripoli, Libya

**Keywords:** Cardiac mass, Cardiac thrombus, Right atrial myxoma, SARS-CoV-2, Post-Acute COVID-19 syndrome (PACS), Case report.

## Abstract

Post-Acute COVID-19 syndrome (PACS) is considered to be one of the least understood post-infectious syndromes. We report a case of a 21-year-old female who had a history of SARS-CoV-2 infection and presented with a right atrioventricular thrombus associated with pulmonary embolism and thrombocytopenia. At the time of admission, she was not vaccinated against SARS-CoV-2, and her serological tests for IgG and IgM antibodies against SARS-CoV-2 were positive. The size of the thrombus measured approximately 6 × 8 × 4 cm, which also led to tricuspid valve insufficiency due to mechanical dilatation of the valve’s ring. The right atrioventricular thrombus also extended up to the inferior vena cava, leading to mild congestive hepatomegaly. Moreover, during thrombectomy, the mass of the thrombus was attached to the interseptal right atrial wall. The histopathological assessment of the core mass revealed that it was a right atrial myxoma hidden inside that large thrombus. We suspect that the formation and propagation of the thrombus to that size occurs as a part of Post-Acute COVID-19 syndrome (PACS). This study reviews and discusses coronavirus disease 2019-relate to thrombus formation inside cardiac chambers in case of a cardiac tumor, like myxoma in the setting of post-acute phase COVID-19 syndrome.

**Supplementary Information:**

The online version contains supplementary material available at 10.1186/s13019-022-01998-1.

## Introduction

SARS-COV-2 is a serious respiratory disease that results from infection with a new coronavirus. As SARS-CoV-2 infection has reached a global scale. Some patients experience multi-organ symptoms and difficulties that last longer than acute illness[1, 2]. According to the Centers for Disease Control (CDC) and National Institute for Health & Care Excellence (NICE) guidelines, the occurrence of symptoms significantly differed according to age and sex. However, the symptoms may last weeks or months after the infection has gone. These circumstances are called Post-Acute COVID-19 syndrome (PACS) or long COVID-19 [3, 4]. This health condition could be worsened in people who have comorbidities. There is evidence that COVID-19 may impact thrombus composition and resistance to thrombolysis [5, 6]. People who have underlying cardiovascular disease are at a higher risk of developing complications. One of the catastrophic complications is thrombosis and clot formation[7]. A thrombus could be formed inside many structures, such as arteries, veins, or even a thrombus inside the heart. Cardiac thrombi are indeed difficult to differentiate from other cardiac tumors, such as myxoma. In the setting of cardiac thrombi, the one presented on the right side of the heart is very rare, and some types of right heart thrombi are challenging to distinguish from a myxoma [8]. Myxoma is the most common cardiac tumor, which emerges from different parts of the heart. They are most commonly found in the left atrium, followed by a right atrium, and rarely in the left ventricle. The right atrium is home to 20% of all sporadic cardiac myxomas. A central or peripheral embolism or intracardiac blockage may cause symptoms in approximately half of the myxoma patients, although the remaining 10% may be entirely asymptomatic [9, 10].

In the current study, a patient with a right atrial myxoma had a huge cardiac thrombus located on the right side of the heart which obliterates approximately the whole right-sided cardiac cavities. Moreover, the patient has a history of SARS-CoV-2 infection 4 months before the presentation. We suspect that this is one of the sequela of (PACS).

## Case presentation

In January 2021, a 21-year-old Libyan female was hospitalized in a tertiary care center in Tripoli, Libya, in critical condition. The patient was admitted to the Cardiac Care Unit (CCU) with difficulty breathing on exertion, dry cough for four months accompanied by a recent history of retrosternal chest pain, paroxysmal nocturnal dyspnea, and orthopnea associated with generalized fatigability. Interestingly, four months prior to admission, she reported breathlessness, dry cough, myalgia, and arthralgia along with intermittent fever reaching up to 40°Cْ. The patient was suspected of having SARS-COV-2 when she presented with positive serological IgG and IgM. It is worth noting that the patient did not have a vaccination against SARS-CoV-19.

In the first exam, the patient was dyspneic at rest, tachypneic, bilateral decreased air entry with no added sound, and abdominal examination showed a distended abdomen with no tenderness or organomegaly. Her Cardiac examination showed muffled heart sounds with no clear murmur heard, Moreover, the patient’s an irregular pulse with a rate of 111 beats /minute, blood pressure was 100/80 mmHg, O2 saturation was 95%, and the temperature was 38.2 °C. Further assessment with a trans-thoracic echocardiogram (TTE) revealed a large thrombus/mass occupying the right atrium and right ventricle that measured 8 × 6 cm.

CT pulmonary angiography revealed a left inferior lobar pulmonary embolism. Despite no duplex ultrasound on the lower limb having been performed, clinical assessment excluded the presence of deep venous thrombosis. Abdominal ultrasound showed only minimal ascites with mild hepatomegaly. While being there in the CCU for approximately 11 days, the initial aim was to manage the case medically. The patient’s management started with rivaroxaban and heparin infusion under close monitoring (basal numbers of PT 21.6 s, aPTT 45.7 s). However, two days after admission to the CCU, the patient’s hematological parameters dropped with hemoglobin 9.5 g/dl and platelet count 85.5 × 10 mm (basal numbers of hemoglobin 10.43 g/dL, platelet count 111.3 10³/µL). Furthermore, the platelet count continued to drop until it reached 21.6 × 10 mm within the first week; liver function tests (LFT) also revealed high readings. During that time, 4 units of platelets were administered with no significant improvement in her thrombocytopenia.

On 26 January 2022, the general condition of the patient was deteriorating. She became dyspneic at rest, tachypnic and pale, her blood pressure was difficult to measure, with an irregular pulse rate of 140 beats/ min. At that time, her hematological parameters showed platelet count 46 10³/µL, hemoglobin 9.98 g/dL, and homeostasis parameters were PT 17.3 s, aPTT 38.1 s. The patient’s condition continued to deteriorate, so surgery was scheduled for the next day after stabilizing the patient.

**Fig. 1:**
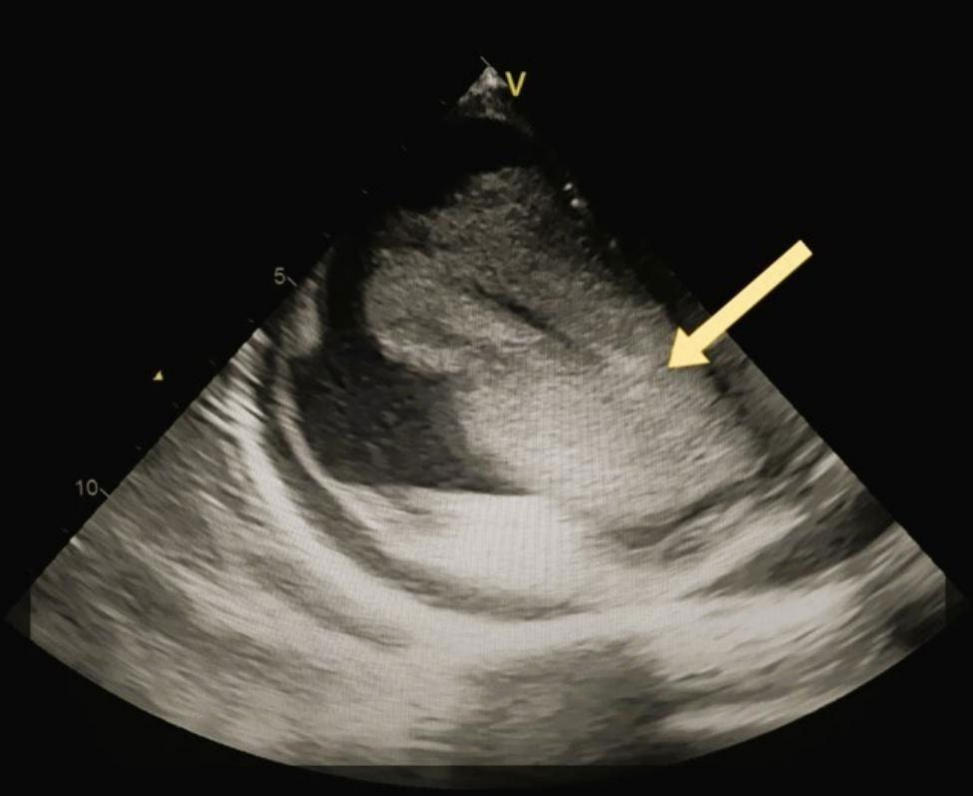
An esophageal view by the trans-thoracic echocardiogram which represents a large atrial thrombus extended to the right ventricle crossing the tricuspid valve

**Fig. 2:**
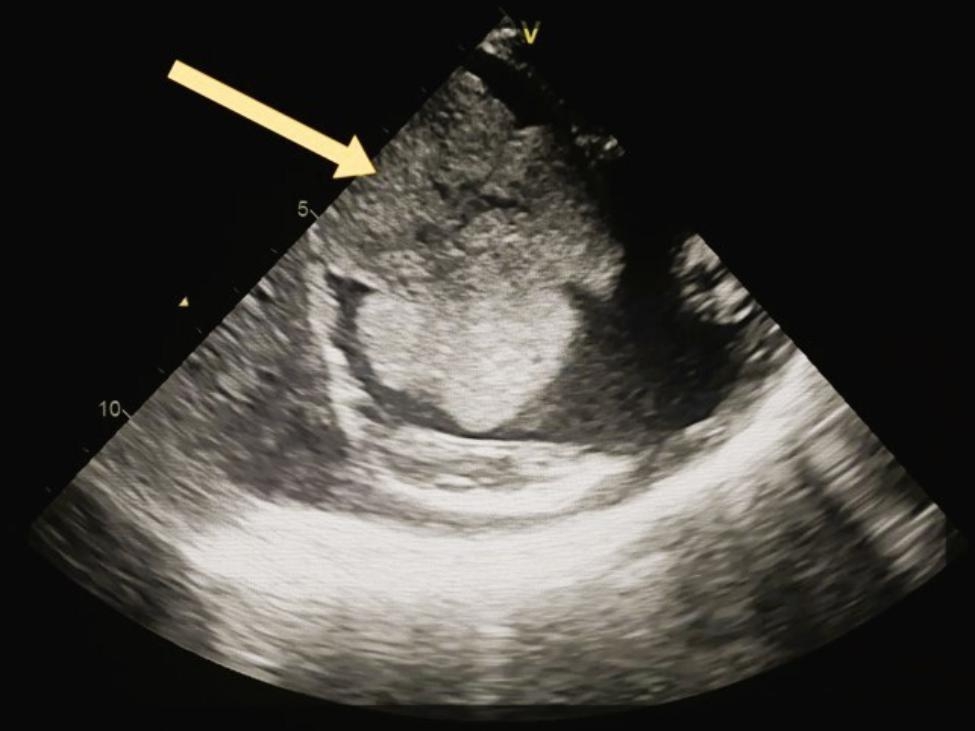
A bicaval view by trans-thoracic echocardiogram which represents a cauliflower mass in the right atrium with a pedicle in the middle, which raised suspicion of myxoma covered with a thrombus

**Fig. 3:**
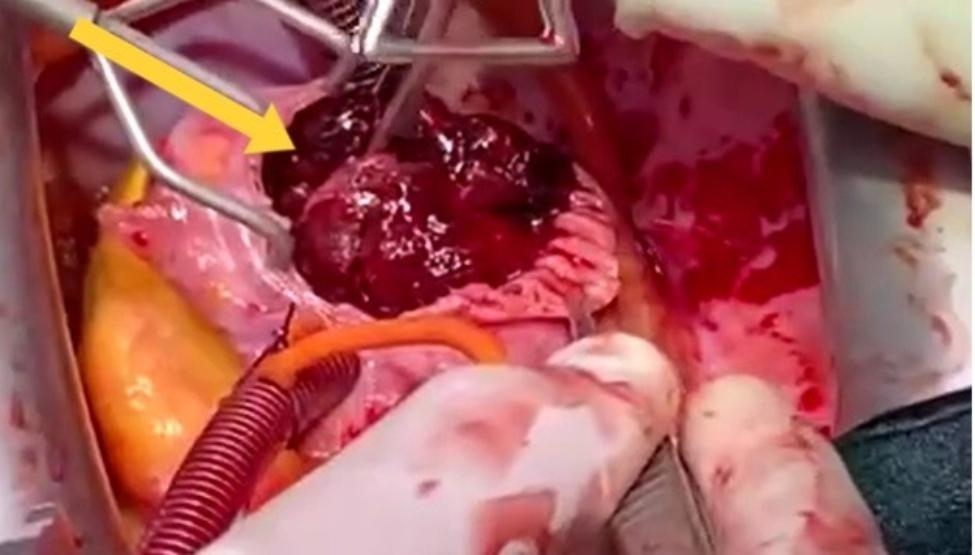
**(a,b)** A thrombus measured 8 × 6 × 4 cm that was located in the right atrium and right ventricle.**Video (1)** Date of surgery: 27 January, 2021. Surgeon: Khaled Masoud Etaleb A clip of open-heart surgery shows right atriotomy, and a red gelatinous mass removed carefully from the right atrium and right ventricle which was extended and attached to at the origin of inferior vena cava

The patient was transferred to the Cardiac Surgery department where a TTE was performed before surgery, which showed a preserved left ventricular contractility with ejection fraction = 51% and TEE revealed a huge atrioventricular thrombus measured 8 × 6 × 4 centimeters (Fig. 1). Furthermore, a cauliflower features and a pedicle in the middle of the thrombus in the right atrium, which raised suspicion of a myxoma covered with a thrombus (Fig. 2). In the operation, the arterial and venous cannulation of the cardiopulmonary bypass (CPB) was inserted, and an additional pulmonary clamp was applied to prevent small thrombi from escaping to the lungs and causing any further pulmonary embolism. Yet, entering the right atrium, a large thrombus occupied the right atrium and right ventricle, and the thrombus extended up to the inferior vena cava (Fig. 3a,b ). However, we encountered difficulties in removing what was suspected to be a thrombus since the mass was connected to the wall of the right atrium with a pedicle, and a specimen was sent for further histopathological assessment. Additionally, extended suction of the inferior vena cava was performed to ensure that no thrombi remained. Tricuspid valve ring annuloplasty was also performed, as the mechanical effect led to valve insufficiency.

Notably, her hemodynamic parameters postoperatively showed a blood pressure of 110/75 mmHg, a pulse rate of 89 beats/min, respiratory rate of 18 per min, and Oxygen saturation of 99%. The central venous pressure at the beginning was 17 cm H2O then decreased to 11 cm H2O after a few hours (Table 2). The patient’s laboratory investigations showed much improvement postoperatively (Table 3). Overall, the operation went smoothly with no complications. She was transferred to the ICU, and her stay was uneventful. After a few days, the patient had significant clinical improvement. The patient was discharged after one -week postoperatively in good general condition under a prophylactic dose of warfarin. During follow-up, the patient complained of having difficulty doing daily tasks along with mild breathlessness, especially with exertion. The TTE after 6 months showed no signs of recurrence of myxoma or right-sided hypertrophy; however, no values of estimated systolic pulmonary artery pressure were recorded to rule out pulmonary hypertension.

**Table:** Table 1

laboratory	values	laboratory	values
Cardiac enzymes	normal	D-dimer	-
hemoglobin	10.43 g/dL	Total Bilirubin	1.650
Platelet count	111.3 10³/µL	ALT	39.2 U/L
WBC count	10.77 10³/µL (neutrophil 62%,lymphocyte 25%)	AST	113.2 U/L
a PT	21.6 s (INR = 2.26)	Creatinine	0.7 mg/dL
aPTT	45.7 s	Urea	23 mg/dL
CRP	93.4 mg/L	LDL	34.4 mg/dL
Na	129.6 mmol/L	HDL	13.6 mg/dL
K	4.7 mmol/L	TGL	70.6 mg/dL
Cl	104 mmol/L	Glucose	131 mg/dL

**Table 2:** The vital signs of the patient one day before and one day after the surgery:

Vital Signs	One day before Surgery	one day after Surgery
Heart Rate	140 bpm	89 bpm
Blood pressure	un recordable	110/75 mmHg
CVP	-	17 cm H2O
RR	27 min	18 min

**Table 3:** After the procedure, her laboratory panel with hemostasis function showed:

laboratory	values	laboratory	values
WBC count	9.8 10³/µL	Total Bilirubin	2
hemoglobin	10.43 g/dL	Direct Bilirubin	1.8
Platelet count	111.3 10³/µL	ALT	16 U/L
D-dimer	5.13 mg/L	AST	31 U/L
a PT	17.1 s (INR = 1.4)	Creatinine	0.3 mg/dL
aPTT	-	Urea	30 mg/dL
CRP	77.4 mg/L	Total protein	4.7 g/L
Na	134 mmol/L	Serum albumin	3 g/L
K	4.5 mmol/L	LDH	667 U/L
Cl	97 mmol/L	Serum Ferritin	737.6 ng/ml

The histopathology study revealed in some slides the presence of a benign tumor composed of many small blood vessels lined by flat endothelial cells and associated with myxoma lepidic cells, in the form of elongated satellite cells having moderate eosinophilic cytoplasm and bland oval nuclei with inconspicuous nucleoli, arranged singly or in small nests and cords surrounding the blood vessels and embedded within the myxoid stroma. Also, there are extensive hemorrhagic areas, fibrin deposition, granulation tissue formation, and wide areas of ischemic-type necrosis, associated with mixed focal acute and chronic inflammatory cells infiltrate (composed of many small lymphocytes, plasma cells, and neutrophils), with some scattered siderophages and fibrosis. Some slides showed blood elements with the line of Zahn with no evidence of inflammatory cells.

## Discussion

Right atrial myxoma (RAMs) is a rare location of cardiac myxoma and only presents about 17 to 20% of cases of cardiac myxoma compared to other sites in the heart. The majority of cases of myxoma are clinically silent; however, they may also present with a variety of clinical manifestations [11, 12]. A thrombus, a myxoma, or a myxoma covered by a thrombus has a great radiological similarity. For that reason, difficulty can encounter in the process to distinguish myxoma from another cardiac differential diagnosis by using trans-thoracic echocardiography (TTE) [13, 14]. However, as shown in (Fig. 2), when we used trans-esophageal echocardiography (TEE) instead, there was something like a pedicle in the center of the thrombus/mass, which raised the suspicion of the presence of a thrombus covering the myxoma.

Right cardiac thrombus has three types: Type (A) is mainly due to thrombus formed inside the peripheral venous system and transferred to be lodged in the heart, captured in transit, usually it is associated with the presence of concurrent pulmonary embolism. Type (B) thrombi are formed in situ due to cardiac abnormalities contributing to their formation. The last type, type (C), is hard to distinguish from cardiac myxoma. Interestingly, SARS-CoV-2 may also contribute to increased pulmonary vascular resistance, eventually leading to right ventricular dilatation with or without dysfunction with or without imaging evidence for peripheral venous thrombosis. Pulmonary hypertension is also one of the contributing factors to the formation of cardiac thrombi, especially type A [8, 15, 16].

Four months before the presentation, she had a history of intermittent fevers, fatigue, and elevated C-reactive protein (CRP). We assume that this could be explained by two reasons. Although myxoma in rare situations can present with atypical manifestations with constitutional symptoms [6], our main probability is that she contracted SARS-COV-2 infection during that period without being diagnosed. The latter hypothesis was proven by the patient’s serological tests regarding IgM, and the IgG of SARS-COV-2 was positive. Tests for antibody detection have a specificity of 99.6% (95% confidence interval [CI] 99.1–99.9) and a sensitivity of 93.9% (95% CI 86.3– 98.0) for samples taken more than 4 months after symptom onset [17]. Another point is that in January 2021, there were no vaccination campaigns in the whole country until late March 2021 [18].

The cardiac injury mechanism of SARS-CoV-2 at the cellular level occurs when the virion particles bind to cardiomyocytes via h-ACE2 receptors via the spike protein, causing systemic endotheliitis, immune-mediated cardiac damage, and inflammatory plaque rupture. Moreover, SARS-COV-2 infection activates the coagulation cascade in addition to the endothelial dysfunction and hyper inflammation caused by a cytokine storm. These factors may be prolonged even after the acute phase of infection [19–25].

A new study conducted by Kell et al., [23] declares that approximately 30% of individuals who contracted SARS-CoV-2 infection have had long COVID. As its mechanism is vastly different from SARS-CoV-2, it should be classified as a separate entity of disease. The etiology of PACS at the molecular level is due to the formation of aberrant amyloid fibrin micro-clots that are triggered by the SARS-CoV-2 spike protein. Microclots are also considered a new antigen that can provoke an autoimmune response that can explain some of the PACS. The spike protein of the SARS-CoV-2 virus binds to fibrinogen in the normal coagulation cascade and causes abnormal clots with an elevated pro-inflammatory response, which leads to the formation of thrombi in different parts of the body.

Many factors may play a role in the formation of cardiac thrombus [in situ] on top of atrial myxoma. Local factors, such as, irregular surface of the tumor, bleeding from the myxoma or blood stasis due to the size of the tumor mass might obstruct the blood flow within the chamber. Furthermore, dysrhythmias such as atrial fibrillation can cause blood to stagnate inside the chamber, resulting in thrombus formation [26]. Macroscopically, myxoma has two different forms, either as solid or polypoid myxoma, the latter type is characterized by an irregular surface [28]. A systematic review and a meta-analysis have been conducted to explore factors of embolism in cardiac myxoma cases and found that the irregular surface of the tumor is one of the contributing factors that lead to thromboembolic events in cardiac myxoma patients [27]. Some cases reported hemorrhage or bleeding from the myxoma tumor itself, this tendency to bleed is more common to be found with right atrial myxoma, eventually foci of the hematoma might form an organized thrombus adhered to the surface of the mass [28]. Aside from these local causative factors, there may also be systemic factors like hypercoagulability, since both myxoma and PACS are considered major hypercoagulability states [24, 29]. Along with this, a study reported a case with cardiac thrombus in PACS patients found that the cumulative incidence of total thrombosis for post-discharged COVID-19 patients was up to 2.5% [27].

In conclusion, long COVID or PACS have not been studied in the literature concerning cardiac myxomas, and it is unclear how their presence in the same circumstances can lead to an interlocking effect. We made an assumption that the PACS or long COVID-19 syndrome is the underlying pathology for this presentation. It will be necessary for future studies to address whether thrombus could form over cardiac myxomas for cases who are having post-acute COVID-19 syndrome. This should significantly improve the treatment and prevention of thrombosis associated with COVID-19 and other cardiovascular complications in the near future.

## Electronic supplementary material

Below is the link to the electronic supplementary material.

Supplementary Material 1

## Data Availability

No Availability of data and materials.
